# The Relationship Between Soleus H-Reflex Following Standing GVS and Postural Control Responses on Firm and Foam Surfaces: An Exploratory Study

**DOI:** 10.3390/brainsci15020115

**Published:** 2025-01-25

**Authors:** Tsubasa Mitsutake, Takanori Taniguchi, Hisato Nakazono, Tomoyuki Shiozaki, Hisayoshi Yoshizuka, Maiko Sakamoto

**Affiliations:** 1 Clinical Research Center, Saga University Hospital, Saga 849-8501, Japan; 2 Department of Physical Therapy, Faculty of Medical Science, Fukuoka International University of Health and Welfare, Fukuoka 814-0001, Japan; taniguchi@takagigakuen.ac.jp (T.T.); yoshizuka@takagigakuen.ac.jp (H.Y.); 3 Department of Occupational Therapy, Faculty of Medical Science, Fukuoka International University of Health and Welfare, Fukuoka 814-0001, Japan; nakazono@takagigakuen.ac.jp; 4 Department of Otolaryngology-Head and Neck Surgery, Nara Medical University, Nara 634-8521, Japan; shiozaki@naramed-u.ac.jp; 5 Education and Research Centre for Community Medicine, Faculty of Medicine, Saga University, Saga 849-8501, Japan; masaka@cc.saga-u.ac.jp

**Keywords:** lateral vestibulospinal tract, galvanic vestibular stimulation, H-reflex, body control response, surface

## Abstract

**Background**: The vestibular postural control system affects standing stability on an unstable surface. However, it is unclear whether maintaining a standing position on different surfaces alters lateral vestibulospinal tract (LVST) excitability and body control responses following vestibular stimulation. This study aimed to investigate the relationship between the soleus H-reflex following galvanic vestibular stimulation (GVSH), a measure of LVST, and post-stimulus body movement responses while standing with eyes closed on different surfaces. **Methods:** Twelve healthy volunteers (mean age 20.4 ± 0.5 years, 7 females) performed eyes-closed standing GVSH on firm and foam surfaces. Body control responses in each condition were evaluated using an inertial measurement unit to monitor neck and pelvic movements, along with surface electromyography to assess muscle activity in the tibialis anterior and soleus muscles. Body responses to the GVS were averaged over up to a second after tibial nerve stimulation. **Results:** We observed a significant negative correlation between the H-wave amplitude of the GVSH on the firm surface and the tibialis anterior muscle activity following stimulation (r = −0.666, *p* = 0.018). No significant differences were observed during the eyes-closed standing GVSH on either firm or foam surfaces (*p* = 0.568). **Conclusions:** Postural maintenance in response to vestibular stimulation may contribute to body stability by regulating tibialis anterior muscle contraction via the LVST. Our findings may help elucidate the neural activity of vestibular function-related standing postural control responses.

## 1. Introduction

Vestibulospinal tracts play a crucial role in postural control. Specifically, the lateral vestibulospinal tract (LVST) descends primarily ipsilaterally through the spinal cord and modulates anti-gravity muscle activity via intraspinal circuits [1,2]. The LVST regulates muscle activity in sitting and standing positions, contributing to the maintenance of a stable posture. LVST excitability affects postural stability on unstable surfaces, which requires vestibular function [3]. Therefore, an accurate assessment of LVST excitability is important for detecting the fall risk caused by vestibular-related balance disorders.

LVST excitability is assessed using the soleus (SOL) H-reflex following square-wave pulse galvanic vestibular stimulation (GVSH) [4,5,6], a method known for its high test–retest reliability [6]. Short-duration galvanic vestibular stimulation (GVS) alters the firing rate of primary vestibular neurons in a polarity-dependent manner [7,8]. A previous study demonstrated that the GVSH has a higher rate of maximum amplitude facilitation in the sitting position than in the prone and supine positions [2]. However, few studies have measured H-wave amplitude with GVSH in the standing position [9], and physical control responses following GVSH have not been evaluated. Given that patients with bilateral vestibular failure and those who have experienced a stroke exhibit absent or reduced somatic sway responses following GVS [10,11], we hypothesized that the H-wave amplitude during standing GVSH is directly related to the sway induced by vestibular stimulation. Clarifying this relationship could provide insight into the neural basis of vestibular input-induced body responses in a fall-prone standing position.

Accurate body responses to standing on different surfaces are important for controlling posture. Standing on a foam surface with the eyes closed is typically used for vestibular training and postural stability assessment [12,13]. Given that standing posture is maintained by enhancing the reliance on vestibular function in sensory strategies [14], standing on a foam surface with eyes closed may increase LVST excitability. A previous study demonstrated that supine GVSH increases LVST excitability after eyes-closed standing training on a soft surface [15]. However, to date, no direct validation experiments have been conducted on the relationship between the ability to maintain the eyes-closed standing position and LVST excitability during standing GVSH on a foam surface. Measuring GVSH while standing may provide useful information regarding the mechanism of postural control involving the vestibular function.

The SOL H-reflex used in GVSH is influenced by potential muscle activity. The peak-to-peak amplitude of the H-reflex is depressed in the upright position compared to that in the supine position [16]. The reduced H-reflex in standing is thought to protect against excessive motor drive for postural maintenance [17,18]. This phenomenon is likely to be influenced by presynaptic inhibition mechanisms at the cortical and/or spinal levels [19,20,21]. Therefore, it is important to evaluate muscle activity using surface electromyography (EMG) before H-reflex measurements. Additionally, clarification of the intra-individual variability in standing H-wave amplitude may validate changes in LVST excitability due to eyes-closed standing GVSH.

GVSH is a quantitative assessment method based on neural mechanisms. Clarifying the body’s response to standing GVSH from a neurophysiological perspective will provide useful information for the clinical application of the LVST assessment method. Changes in LVST excitability on different floor surfaces play an important role in understanding the vestibular postural control system. This study aimed to explore the relationship between the standing GVSH amplitude and postural sway response following GVS in an exploratory manner. Additionally, this study examined GVSH amplitudes while standing with eyes closed on firm and foam surfaces and tested background EMG and H-wave amplitude internal variability.

## 2. Materials and Methods

### 2.1. Participants

Twenty healthy volunteers (mean age 20.5 ± 0.7 years, 12 females) participated in this study. The participants had no history of neurological or orthopedic diseases, vertigo, or other vestibular dysfunctions that could affect their standing postural control. During enrollment, participants who were unable to hold the standing position when a stimulus duration of 200 ms was applied at 3 mA GVS in the closed-eye standing position on a soft floor were excluded from this study.

This study was approved by the Ethical Review Committee of the Fukuoka International University of Health and Welfare (approval number: 20-fiuhw-011). The study was conducted in accordance with the principles and guidelines of the Declaration of Helsinki. All participants provided written informed consent after being informed of the nature and purpose of this study.

### 2.2. Experimental Procedures

**Figure 1:**
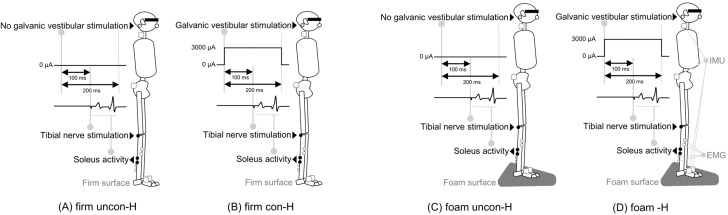
Experimental procedure. Participants were randomly assigned to the following four conditions: (**A**) unconditioned H-reflex on firm surface (firm uncon-H), (**B**) conditioned H-reflex following GVS on firm surface (firm con-H), (**C**) unconditioned H-reflex on foam surface (foam uncon-H), and (**D**) conditioned H-reflex following GVS on foam surface (foam con-H). All conditions were measured by the body sway of the neck and pelvis using IMU and the muscle activity of the right tibialis anterior and right soleus muscles using surface EMG. IMUs in both feet were used to define body position. Conditions (**B**,**D**) involved GVS at a stimulus intensity of 3000 µA for 100 ms prior to tibial nerve stimulation to elicit the soleus H-reflex. Abbreviations: GVS, galvanic vestibular stimulation; IMU, inertial measurement unit; EMG, electromyography.

We measured the unconditioned H-reflex (uncon-H) without GVS and the conditioned H-reflex (con-H) following GVS while standing with eyes closed on firm and foam surfaces. All participants performed the four conditions: uncon-H on firm surface (firm uncon-H; Figure 1A), con-H on firm surface (firm con-H; Figure 1B), uncon-H on foam surface (foam uncon-H; Figure 1C), or con-H on foam surface (foam con-H; Figure 1D). The order of measurement for each condition was randomly assigned. All conditions were assessed for neck and pelvic body movements using an inertial measurement unit (IMU), and for muscle activity in the right tibialis anterior muscle (TA) and right SOL using surface EMG.

### 2.3. Preparation for Soleus H-Reflex Measurement

Before measuring the standing GVSH, the participants were assessed in the supine position with the right SOL H-reflex using a Neuropack (Nihon Kohden, Tokyo, Japan). The right foot was fixed with an orthotic to minimize ankle joint movement, and the joint position was set at 0° plantar dorsiflexion and approximately 15° knee flexion. The participants were instructed to keep their heads and upper extremities in a stable position and their eyes closed while awake during the experiment [5,22]. Right SOL activity was recorded using bipolar Ag/AgCl electrodes placed 2 cm apart in the center of the muscle belly. The nerve-stimulating electrode was fixed to the tibial nerve using a belt-type electrode. The ground electrode was placed on the mediolateral portion of the stimulating and exploratory electrodes on the same leg. The electromyographic signals were amplified using a computerized modular electromyographic detection unit with a 15–3 kHz bandpass filter, converted to a digital signal, and stored on a personal computer. To adjust the ascending limb of the H-reflex recruitment curve, this study measured the right SOL H-reflex amplitude at stimulus levels that produced M-waves corresponding to 15–25% of the maximum M-wave [5,9].

Subsequently, the present study measured the amplitudes of the M- and H-waves while standing with eyes closed as a pre-test and compared these ratios to those measured in the supine position. If significant changes in the M- or H-wave amplitudes were observed, the stimulation site and intensity were reconfirmed.

### 2.4. Standing GVSH Measurement

To assess LVST excitability, we performed GVS 100 ms before tibial nerve stimulation to elicit the SOL H-reflex. GVS was performed with square-wave stimulation using a DC-Stimulator-Plus (NeuroCare Group GmbH, Munich, Germany), with stimulation control managed via a personal computer running DC-StimEditor (Medical Try System, Tokyo, Japan). The GVS electrode was a 3 × 3 cm rubber electrode with the cathode affixed to the right mastoid process and the anode to the left mastoid process. To reduce the impedance of the electrodes affixed to both the mastoid processes and the right leg, the skin was cleaned with alcohol and an exfoliating cleanser, and an electrode gel was applied. The impedance of the GVS was kept below 10 kΩ. Prior to testing, participants were instructed to hold a standing position with their eyes closed, legs closed, and head facing forward to confirm that the GVS did not produce pain behind the eyes or moodiness and that body sway appeared in the anodal direction [23].

H-reflex measurements were conducted 10 times randomly at 7–13 s intervals to prevent stimulus adaptation with a stimulus duration of 1 ms. The change rate of the H-reflex to assess LVST excitability was calculated using the peak-to-peak amplitude, following this formula: (con-H amplitude/uncon-H amplitude −1 × 100%) [3,6], and GVSH was recorded on both the firm (firm GVSH) and foam surfaces (foam GVSH). The H-reflex for each condition was determined by averaging 10 measurements.

### 2.5. Body Control Response Measurement

Body control responses were assessed by measuring body sway with IMUs (Myomotion, Noraxon USA Inc., Noraxon, Scottsdale, AZ, USA) and muscle activity with surface EMG (Clinical DTS, Noraxon USA Inc., Noraxon, Scottsdale, AZ, USA). We synchronized the Neuropack, IMUs, and surface EMG to ensure consistent timing of the measurements.

For body sway measurements, IMUs were placed on the C7 spinous process (neck), the middle of the pelvis, and the back of both feet based on anatomical landmarks. Both foot IMUs were used to define the body position. Linear acceleration data from each sensor were recorded along the vertical (VT), mediolateral (ML), and anteroposterior (AP) directions. To account for body sway, the root mean square (RMS) of each direction was extracted, and the postural stability value was calculated as the RMS sway. All measurements were recorded at a sampling frequency of 1500 Hz.

Surface EMG signals were acquired using an 8-channel wireless electromyography system (Clinical DTS, Noraxon USA Inc., Scottsdale, Scottsdale, AZ, USA) with 16-bit resolution and a common mode rejection ratio of >100 dB. After appropriate skin preparation, four circular Ag/AgCl surface electrodes (electrode diameter, 34 mm; distance between electrodes, 30 mm) were placed—two on the right TA and two on SOL—following the recommendations of the Surface EMG for Non-Invasive Assessment of Muscles [24]. The electrode on the SOL was affixed such that it did not overlap with the H-reflex measurement position. The raw signals were pre-amplified 1000 times and sampled at 1500 Hz using a 500 Hz low-pass filter.

After measuring postural control responses under all conditions, the maximum voluntary contraction (MVC) of each muscle was measured for 5 s using manual resistance. Each MVC test was repeated three times [25,26]. To measure the MVC of each muscle, the participants were seated with their knees fully extended and their feet in a neutral position between dorsiflexion and plantar flexion. To measure the MVC of the SOL, participants were instructed to perform maximal isometric ankle plantar flexion for 5 s. For TA testing, participants were instructed to perform 5 s of maximal isometric ankle dorsiflexion with the examiner’s hand placed on the dorsum of the foot. Verbal encouragement was provided between muscle contractions using standard scripts.

### 2.6. Data Extraction and Processing

The IMU and surface EMG data were processed using analysis software (MyoResearch 3, Noraxon, AZ, USA). The IMU data were filtered using a 10 Hz IIR high-pass filter, while the surface EMG data were filtered using a 20–450 Hz FIR bandpass filter. Both data sets were analyzed using RMS with a 100-smoothing window.

Background EMG was RMS averaged over the range from the SOL EMG activity to 100 ms before each tibial nerve stimulation in the firm uncon-H, firm con-H, foam uncon-H, and foam con-H conditions. The post-H-reflex EMG was calculated by averaging the TA and SOL muscle activities up to 1 s after tibial nerve stimulation for each condition. Similarly, post-H-reflex IMU data were obtained by averaging RMS values in the VT, ML, and AP directions for the neck and pelvis up to 1 s after tibial nerve stimulation.

For each muscle, we randomly extracted 3 s EMG amplitudes from the three MVC trials and averaged them. The %MVC was then calculated by normalizing the background and post-stimulus EMG values for each standing condition.

### 2.7. Statistical Analysis

Statistical analyses were performed using several methods. First, we confirmed the normality of the data using the Shapiro–Wilk test. A paired *t*-test was used to check for significant differences in the H-wave amplitude between the firm and foam GVSH conditions. Second, a correlation analysis was conducted to examine the relationship between the H-wave amplitude of the GVSH on the firm and foam surfaces and the post-stimulus EMG of the TA and SOL, as well as the RMS sway values in the VT, ML, and AP directions for the neck and pelvis. Third, the coefficient of variance (CV) of the H-wave amplitude was calculated across 10 trials for each individual to confirm the variability of the H-wave in each condition. A two-way analysis of variance (ANOVA) was conducted to identify significant differences in the CV of the H-wave amplitude between the surface (firm, foam) and stimulus (uncon-H, con-H) conditions. Finally, another two-way ANOVA assessed significant differences in the background EMG of SOL between the surface (firm, foam) and stimulus (uncon-H, con-H) conditions. If significant interactions were found, simple main effect tests and post hoc analyses with Bonferroni corrections were conducted to evaluate multiple comparisons.

Effect sizes were calculated using Cohen’s *d* for *t*-tests and partial eta-square (η_p_^2^) for ANOVA. Statistical analyses were performed using IBM SPSS^®^ Statistics for Windows version 26 (IBM Corporation, Armonk, NY, USA). Statistical significance was set at *p* < 0.05.

## 3. Results

A total of 12 participants (mean age 20.4 ± 0.5 years, 7 females) were included in this study, and eight were excluded: five could not stand with their eyes closed during GVS on a foam surface, and three were unable to focus on the ascending limb of the standing H-reflex.

**Figure 2:**
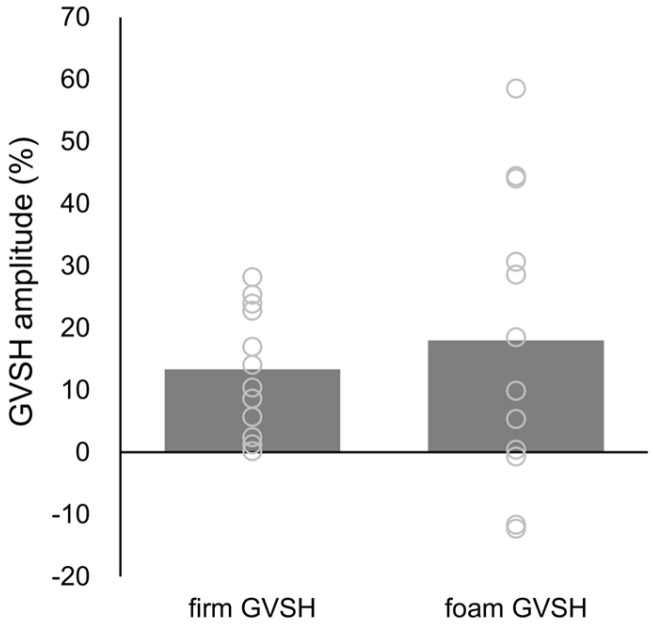
H-wave amplitude of GVSH during eyes-closed standing on firm and foam surfaces. Gray circles indicate individual values, and gray bars indicate average values. Abbreviation: GVSH, H-reflex following galvanic vestibular stimulation.

**Figure 3:**
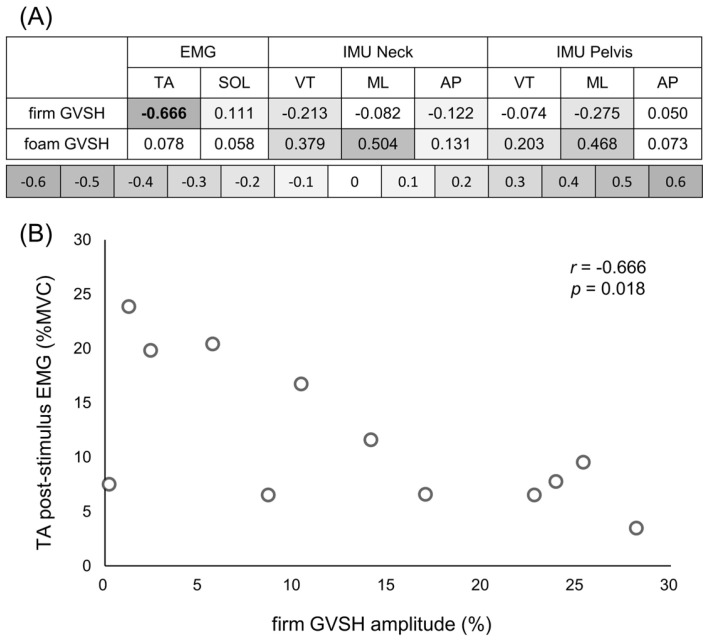
(**A**) Correlation coefficients between GVSH and post-GVS tibialis anterior, soleus EMG activity, and RMS motion of the neck and pelvis in the vertical, mediolateral, and anteroposterior directions. Dark gray circles indicate strong correlation coefficients between GVSH and body control responses. (**B**) Scatter plots show the relationship between firm GVSH amplitude and tibialis anterior EMG activity after GVS. Abbreviations: GVSH, H-reflex following galvanic vestibular stimulation; GVS, galvanic vestibular stimulation; EMG, electromyography; RMS, root mean square; IMU, inertial measurement unit; VT, vertical direction; ML, mediolateral direction; AP, anteroposterior direction; TA, tibialis anterior muscle.

No significant difference was observed in H-wave amplitude in the standing GVSH, which was 13.33 ± 10.02% on the firm surface and 17.99 ± 23.30% on the foam surface (t = −0.589, *p* = 0.568, d = 0.230) (Figure 2). The relationship between the GVSH amplitude and body control response values is presented in Figure 3A. A significant negative correlation was observed between the firm GVSH amplitude and the post-stimulus EMG of the TA (r = −0.666, *p* = 0.018) (Figure 3B). There were no significant correlations in other parameters.

**Figure 4:**
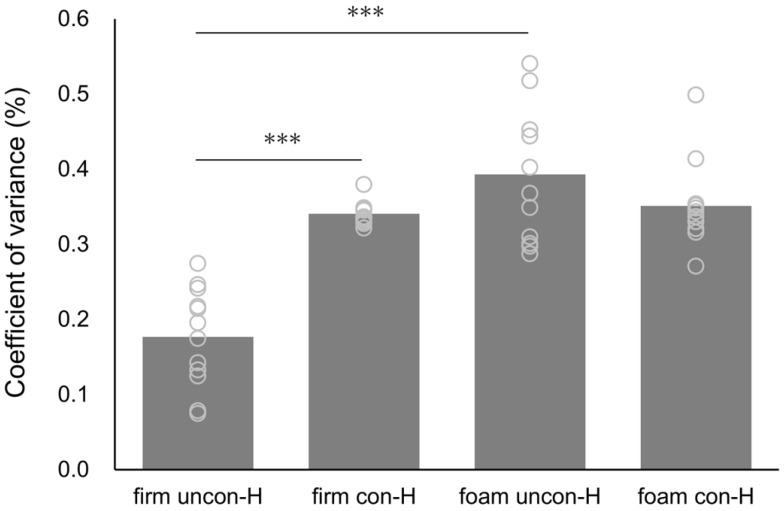
Coefficient of variance for H-wave amplitude in each eyes-closed standing condition. Gray circles indicate individual values, and gray bars indicate average values. Abbreviations: firm uncon-H, unconditioned H-reflex on a firm surface; firm con-H, conditioned H-reflex following galvanic vestibular stimulation on a firm surface; foam uncon-H, unconditioned H-reflex on a foam surface; foam con-H, conditioned H-reflex following GVS on the foam surface. *** *p* < 0.001.

**Figure 5:**
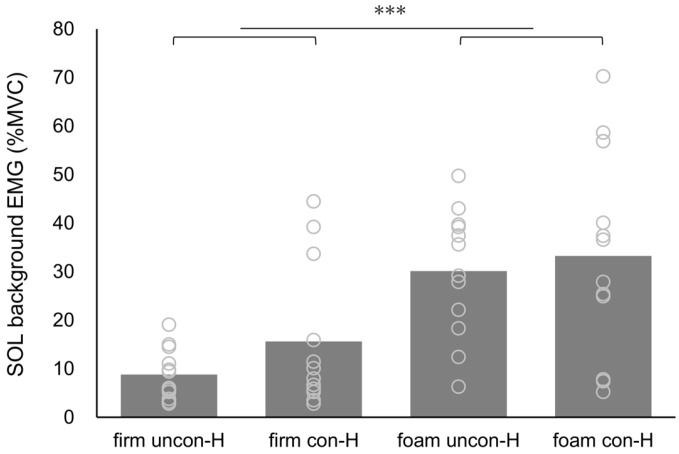
Background EMG activity of the soleus muscle before H-reflex measurement in each eyes-closed standing condition. Gray circles indicate individual values, and gray bars indicate average values. Abbreviations: SOL, soleus muscle; EMG, electromyography; firm uncon-H, unconditioned H-reflex on a firm surface; firm con-H, conditioned H-reflex following galvanic vestibular stimulation on a firm surface; foam uncon-H, unconditioned H-reflex on a foam surface; foam con-H, conditioned H-reflex following GVS on the foam surface. *** *p* < 0.001.

The H-wave CV and SOL background EMG values, along with the results of the two-way ANOVA, are presented in Table 1. The H-wave CV demonstrated a significant interaction for the surface and stimulus conditions (F = 36.934, *p* < 0.001, η_p_^2^ = 0.771) as well as significant main effects for the surface condition (F = 53.870, *p* < 0.001, η_p_^2^ = 0.830) and stimulus condition (F = 12.161, *p* = 0.005, η_p_^2^ = 0.525). Regarding the main effect, the CV in the surface condition was significantly higher on the foam surface than on the firm surface (*p* < 0.001), and the CV in the stimulus condition was significantly higher for con-H than for uncon-H (*p* = 0.005) (Figure 4). Moreover, the uncon-H condition demonstrated significantly higher CV values on the foam surface than on the firm surface (*p* < 0.001). Additionally, the firm surface condition demonstrated significantly higher CV values for con-H than for uncon-H (*p* < 0.001) (Figure 4). SOL’s background EMG showed a significant main effect of surface condition (F = 20.390, *p* < 0.001, η_p_^2^ = 0.650), with significantly higher values for the foam surface than for the firm surface (*p* < 0.001) (Figure 5). No significant differences were observed in the other parameters.

A post hoc test was performed on the validity of the sample size using the G*Power software (version 3.1.9.7). Based on the results of this study (r = −0.666, significance level 0.05, and total sample size of 12), the calculated power was 0.8.

## 4. Discussion

No significant difference in the H-wave amplitude was observed between firm and foam GVSH, indicating no change in LVST excitability across different surfaces. Generally, standing on a foam surface with eyes closed seems to prioritize sensory strategies that rely heavily on vestibular information by limiting visual and somatosensory inputs. However, postural control strategies involving vestibular sensory information may vary based on the individual’s sensory reliance. There is a subset of non-responders to vestibular stimuli who place low emphasis on vestibular sensation [27,28]. Participants with lower reliance on vestibular function might compensate by using somatosensory information to maintain a stable standing position. The lack of significant differences in the GVSH across different surfaces in this study may be due to individual variations in sensory preference. From another perspective, the LVST might have been excited regardless of the surface conditions. There is still a limited understanding of how the convergence of signals from the descending pathways in the spinal cord and the integration of descending motor commands influence ongoing movements [29]. Given that a previous study has shown that vestibular nerve stimulation with weak electrical currents enhances postural stability while standing on a firm surface [30], it is possible that LVST excitation in this study was similarly activated on both firm and soft surfaces during eyes-closed standing.

This study yields several noteworthy results, including a marked negative correlation between firm GVSH amplitude and EMG activity in the TA muscle following stimulation. This suggests that increased LVST excitability in the upright limb position is related to the suppression of ankle dorsiflexor activity after GVS. The LVST excites extensor motoneurons and suppresses flexor motoneurons via spinal interneurons in animal experiments [29,31,32], which is consistent with previous research. The vestibulospinal tract plays a crucial role in controlling standing posture and adapting to various daily activities. Postural maintenance in response to vestibular stimulation may contribute to body stability by regulating TA muscle contraction. Our findings suggest that LVST plays a crucial role in controlling body function.

In contrast, no significant correlation was observed between the firm GVSH amplitude and SOL EMG activity in the present study. Given that the LVST excites motoneurons in the extensor muscles [29], we predicted a positive correlation between standing GVSH amplitude and SOL EMG. These unexpected results might be attributed to the role of muscle activity in controlling posture in the TA and SOL. The SOL, with a high percentage of type I fibers, plays a key role in maintaining antigravity positions. In contrast, the TA, which has a high percentage of type II fibers, is involved in stabilizing through fast muscle contractions [33]. Postural control responses were evaluated after vestibular stimulation that induced body sway. Therefore, our results may have been influenced by the fast muscle contraction of the TA, which involves reactive postural control through ankle-joint strategies in response to stimulation.

No significant correlation was observed between standing GVSH amplitude and IMU measurements of the neck or pelvis in the present study. Postural maintenance following vestibular stimulation is influenced by various postural control systems. The lack of significant relationships with the IMU data may be due to differences in body control strategies among individuals.

Regarding the reproducibility of the standing H-reflex, the H-wave CV was considerably higher in the foam condition than in the firm condition. Similar results were observed for the SOL background EMG. The adjustment of the standing SOL H-reflex amplitude is influenced by the adaptation of the ankle strategy to the standing condition [34]. A previous study demonstrated that standing on a foam surface increased ankle joint motion more than standing on a firm surface [14]. The TA and SOL activities contribute to postural stability through an ankle-joint strategy of repetitive fine muscle contractions to maintain standing on a foam surface. The amplitude of the standing H-reflex varies with the phase-dependent modulation of the measured muscle activity, influenced by changes in foot pressure [35]. In other words, the H-wave amplitude may be reduced by hypercontraction of the SOL at the time of tibial nerve stimulation. Increased standing H-wave CV and SOL background EMG activity suggest large interindividual variability in foam GVSH. It is possible that these results might have led to the lack of significant differences in the GVSH amplitude owing to different floor surfaces. Future studies should investigate whether controlling background EMG activity produces different results for LVST assessments on foam surfaces.

The H-wave CV was significantly higher for con-H than for uncon-H on firm surfaces. This suggests that GVS may reduce the reproducibility of standing H-waves. Since the LVST plays a role in controlling standing posture, the standing GVSH measurement has the advantage of directly assessing vestibular nerve activity. However, to improve the reproducibility of standing GVS assessments, it is important to adjust the stimulus timing and intensity.

The present study has some limitations. The SOL background EMG was calculated as a simple EMG average of 100 ms before tibial nerve stimulation, and the scope of background EMG analysis included body control responses by GVS and M-wave amplitude. Previous studies have analyzed the co-contraction index of the TA and SOL [36] and reciprocal agonist–antagonist muscle activity [37]. Future studies should utilize more detailed analytical methods and measure EMG before GVS to verify whether background EMG affects standing GVSH. Second, the present study did not examine the relationship between standing uncon-H and post-stimulus postural control responses owing to high interindividual variability in the H-reflex. To further investigate the neural mechanisms underlying standing GVSH, it is necessary to increase the number of participants and analyze both standing uncon-H and con-H amplitudes. Finally, the number of participants in this study was reduced due to the study design requiring high postural control skills. The validity of this study could be enhanced by characterizing the excluded participants. Factors contributing to interindividual variability in GVS may be elucidated by identifying potential confounding factors, including sensory strategies and spatial awareness abilities.

Despite these limitations, the present study suggests that a firm GVSH amplitude may reduce TA EMG activity after stimulation. To the best of our knowledge, this is the first study to investigate the relationship between LVST excitability and body control responses after vestibular stimulation. Furthermore, this study validated the changes in LVST excitability induced by foam GVSH. These findings may provide evidence that LVST excitability varies according to standing posture and individual sensory weight.

## Figures and Tables

**Table 1:** Results of two-way analysis of variances for surface and stimulation for H-wave CV and SOL background EMG.

	Firm	Foam	Surface			Stimulation			Surface × Stimulation		
			*F*-Value	*p*-Value	η_p_^2^	*F*-Value	*p*-Value	η_p_^2^	*F*-Value	*p*-Value	η_p_^2^
H-wave CV											
uncon-H	0.18 ± 0.07	0.39 ± 0.09	53.870	**<0.001**	0.830	12.161	**0.005**	0.525	36.934	**<0.001**	0.771
con-H	0.34 ± 0.01	0.35 ± 0.06									
SOL background EMG											
uncon-H	8.83 ± 5.27	30.12 ± 13.14	20.390	**<0.001**	0.650	2.633	0.133	0.193	0.428	0.527	0.037
con-H	15.61 ± 14.82	33.25 ± 21.12									

The data are expressed as the means and the standard deviations. Abbreviations: CV, coefficient of variance; SOL, soleus muscle; EMG, electromyography; uncon-H, unconditioned H-reflex alone; con-H, conditioned H-reflex following galvanic vestibular stimulation.

## Data Availability

All data generated or analyzed during this study are included in this published article.
